# Anti-Microbial Biopolymer Hydrogel Scaffolds for Stem Cell Encapsulation

**DOI:** 10.3390/polym9040149

**Published:** 2017-04-22

**Authors:** Philipp T. Kühn, René T. Rozenbaum, Estelle Perrels, Prashant K. Sharma, Patrick van Rijn

**Affiliations:** 1 University of Groningen, University Medical Center Groningen, Department of Biomedical Engineering-FB40, W.J. Kolff Institute for Biomedical Engineering and Materials Science-FB41, A. Deusinglaan 1, 9713 AV Groningen, The Netherlands; p.t.kuhn@umcg.nl (P.T.K.); r.t.rozenbaum@umcg.nl (R.T.R.); e.f.d.perrels@student.rug.nl (E.P.); p.k.sharma@umcg.nl (P.K.S.); 2 University of Groningen, Zernike Institute for Advanced Materials, Nijenborgh 4, 9747 AG Groningen, The Netherlands

**Keywords:** hydrogel, alginate, chitosan, stem cell scaffold, anti-microbial

## Abstract

Biopolymer hydrogels are an attractive class of materials for wound dressings and other biomedical applications because of their ease of use and availability from biomass. Here, we present a hydrogel formation approach based on alginate and chitosan. Alginate is conventionally cross-linked using multivalent ions such as Ca^2+^ but in principle any polycationic species can be used such as polyelectrolytes. Exchanging the cross-linking Ca^2+^ ions partially with chitosan, which at pH 7 has available positive charges as well as good interactions with Ca^2+^, leads to an improved Young’s modulus. This gel is non-toxic to mammalian cells and hence allows conveniently for stem cell encapsulation since it is based on two-component mixing and gel formation. Additionally, the chitosan is known to have a bactericidal effect which is retained when using it in the alginate–chitosan gel formation and the formed hydrogels displayed bactericidal effects against *P. aeruginosa* and *S. aureus*. The combination of anti-bacterial properties, inclusion of stem cells, and the hydrogel nature would provide an ideal environment for complex wound healing.

## 1. Introduction

Wound healing is a complex process, which is often treated using a wound dressing [1,2]. Regular wound dressings only provide a protective barrier against contaminations and are not regarded as sufficient as it is known that wound dressings can modulate, support, and delegate the healing process [3]. Hence the passive bandage is being replaced by novel active wound dressings [4,5,6]. Dressings have been developed to enhance wound healing and combating infections rather than only preventing them [4,7]. These are capable of creating an ideal environment, not just for wound healing, but also for promoting tissue regeneration [4,5,7,8,9,10]. Structured scaffolds such as membranes, electro-spun meshes, and hydrogels are considered as major developments in wound dressings [5,11,12]. A fibrous and “wet” system resembles the natural extracellular matrix to a greater extent, thereby directing cellular processes that enable the wound to heal faster and with a lower chance of scarring.

Materials most frequently used for wound dressing design are biopolymers such as alginate, chitin/chitosan, hyaluronic acid, and gelatin/collagen due to their biocompatibility, low toxicity, biodegradability, or resorption, and their ease of processing [6,7,11,13,14,15]. In particular, alginate and chitosan are known and embraced for their ease of use and the possibility for chemical modifications. Chitosan has the additional feature of being anti-microbial [16,17].

Many systems have been described using either alginate or chitosan but the combination is limitedly used and even less as a hydrogel structure. Alginate–chitosan-based materials have been developed in the form of anti-microbial nanoparticulates [18], microcapsules for drug delivery [19], polyelectrolyte dense membranes [20,21], and polyelectrolyte membrane coatings [22,23]. Recently, it was shown that rehydrated alginate–chitosan films enabled faster wound healing although the preparation method described would not be tolerated by incorporated stem cells. [24] Including stem cells would be of particular interest, as these are considered excellent therapeutic components for promoting wound healing and reducing fibrotic responses [8,9,24,25,26].

Here we developed a two-component mixing approach for creating anti-microbial alginate–chitosan hybrid hydrogels tolerated by human bone marrow-derived mesenchymal stem cells (hBM-MSC). The method can be used for both a preformed dressing system and a wound filling system depending whether wounds are superficial (dermal) or irregularly shaped. The anti-microbial properties of chitosan were tested against *Pseudomonas aeruginosa* (*P. aeruginosa*, Gram-negative, rod-shaped) and *Staphylococcus aureus* (*S. aureus*, Gram-positive, spherical), which are both important microbes in wound infections [27,28].

## 2. Materials and Methods

All chemicals were obtained from Sigma-Aldrich, St. Louis, MO, USA, unless stated otherwise. Alginic acid sodium salt (viscosity of 15–20 cP, 1% in H_2_O) and chitosan (medium molecular weight, viscosity of 200–800 cP, 1 wt % in 1% acetic acid at 25 °C) were used for all experiments. Cell cultures flask and wells plates were purchased from Greiner Bio-one (Münster, Austria). Human bone marrow-derived mesenchymal stem cells (Lonza™) were used for the cell experiments. hBM-MSCs were observed using a LEICA TCS SP2 CLSM (Leica Microsystems B.V., Amsterdam, The Netherlands) equipped with a 40× NA 0.80 water immersion objective. Cell viability was analyzed using an XTT assay (Applichem A8088, Panreac Applichem, Darmstadt, Germany). For anti-microbial studies, *Staphylococcus aureus* ATCC 29213 and *Pseudomonas aeruginosa* ATCC 43392, obtained from wound isolates, were used.

**Gel formation:** Gels were formed by mixing one volume of a 2 wt % alginate solution with one volume of electrolyte solution (chitosan/CaCl_2_). This results in a gel containing 1 wt % alginate. For the electrolyte solution, the overall amount of charge was kept constant. It was calculated that a stock solution of 50 mM CaCl_2_ and a stock solution of 15.7 g/L chitosan, which equals 100 mM of the monomer, formally contain the same amount of positive charge (under the assumption that all amine groups are protonated). The different gels were obtained by mixing the CaCl_2_ and chitosan solution varying the overall charge contribution of chitosan between 0 and 100%, and to reach the same final volume as the desired volume of alginate solution. These were mixed to obtain the final gel.

**Cell encapsulation:** For the cell encapsulation, hBM-MSCs (p7) were suspended in the alginate solution in a concentration of 10^6^ cells/mL, before gel preparation. Alginate solution was prepared using DMEM (Dulbecco’s Modified Eagle Medium, Thermo Fischer Scientific, Waltham, MA, USA) rather than water, which ensured a higher viability of the cells. This solution was then used in a manner similar to that described above for the regular gel preparation. No significant difference in gel properties were found using DMEM instead of water. The medium was refreshed every 2 days. Cells were analyzed after 1 and 5 days of incubation.

**Determining Young’s Modulus:** The stiffness (Young’s modulus) of the gels was measured using LLCT (Low Load Compression Tester, Wipotec Wiege–und positioniersysteme GmbH, Kaiserslautern, Germany). A uniaxial compression was performed measuring strain and stress in a non-destructive way. Gel samples were applied on filter paper resting on a glass slide in order to avoid sample displacement during compression. A few microliters of water were added on top of the sample ensuring contact of the sample with the plunger before commencing compression. All measurements were performed with a fixed strain rate of 5%/s and a maximum deformation of 20%. All measurements were performed in triplicate. Obtained values for stress and strain were extrapolated using Hooke’s Law. For determining stiffness, only the initial 10% of the stress vs. strain curve were taken into account.

**Atomic Force Microscopy:** The gels were imaged using atomic force microscopy (AFM) in terms of morphology. The gels were measured in their hydrated state using an atomic force microscope (AFM) model Dimension 3100 Nanoscope V (Veeco, Plainview, NY, USA) in contact mode and wet state with 0.24 N/m tips. All data were processed using Nanoscope Analysis (Veeco, Version 1.70). Samples were prepared by consecutively adding a drop of alginate solution onto a glass substrate followed by a drop of gelating solution. After gelation, excess liquid was carefully blotted from the side but leaving the gels still in their hydrated form.

**Cell culture hBM-MSCs:** Human bone marrow derived mesenchymal stem cells (hBM-MSC) were used. Cells were incubated at 37 °C, 5% CO_2_ at maximum humidity. Cell culture stock was kept in liquid nitrogen. Cells were counted with hemocytometer. Alpha-MEM complete (10% FBS and 0.1% AA_2_P in Alpha-MEM) was used as the growth medium. Cells were harvested from culture flasks using trypsin for 3–5 min at 37 °C. Cells were cultured at ~80% confluence and 30% was seeded in a new daughter flask.

**Cytotoxicity assay (XTT):** hBM-MSCs were used for determining cytotoxicity. A direct cytotoxicity assay was performed by adding preformed gel directly to the cells. Cells were grown and harvested and seeded in a 96-well plate with a density of 2.5 × 10^4^ cells per well. Cells were incubated for 24 h to ensure sufficient adherence and were washed with PBS prior to the addition of the gel. Cells were incubated for 24 and 120 h together with the gel. The medium was changed every 2 days. XTT and PMS were added to the cells and incubated for 3 h prior to analysis using absorbance measurements at 485 and 690 nm. The absorbance at 480 nm was used for quantifying the metabolic activity observed as the reduction of XTT, while measuring at 690 nm provided the nonspecific absorbance.

**Live/dead staining:** Live/dead staining was performed using PBS containing calcein AM (2 µM, Life Technology) and ethidium homodimer-1 (4 µM, Invitrogen molecular probes) to stain the cells, 30 min prior to microscopy.

**Bacterial culture conditions and harvesting:** *Staphylococcus aureus* ATCC 29213 and *Pseudomonas aeruginosa* ATCC 43392, wound isolates, were taken from a frozen stock, and streaked over a blood agar plate, and incubated for 24 h at 37 °C. A single colony from the plate was taken to inoculate 10 mL of tryptic soya broth (TSB) and incubated for 24 h at 37 °C under static conditions. Two milliliters of these cultures were added to 40 mL of TSB, and again incubated for 18 h at 37 °C at 150 rpm. Bacteria were harvested by 5 min centrifugation at 5000 g, and washed twice with 10 mL of phosphate buffered saline (PBS, 10 mM potassium phosphate, 0.15 mM NaCl, pH 7.0). *S. aureus* ATCC 29213 was sonicated (Vibra cell model 275, Sonics and Material Inc., Danbury, CT, USA) three times for 10 seconds on ice to break bacterial aggregates. Bacteria were counted using the Bürker Türk counting chamber, and diluted in PBS to 3 × 10^8^ colony forming units/mL.

**Gel treatment conditions:** Glass slides were cut in squares of 1 cm × 1 cm, and washed three times with 2% RBS35 (Omnilabo International BV, Breda, The Netherlands) in a sonication bath (Transsonic TP 690, Elma GmbH & Co, Singen, Germany). After the slides were rinsed with ultrapure water and placed in methanol for 5 min, they were washed with ultrapure water, air-dried, and sterilized by autoclaving. Glass slides were placed in Petri dishes and 3 × 10^8^ CFU/mL of either *S. aureus* ATCC 29213 or *P. aeruginosa* ATCC 43392 was added, and incubated at 30 RPM at 37 °C to let the bacteria adhere to the glass slides. After one hour, glass slides were rinsed with PBS and either placed in a 24-well plate (two component treatment) or in Petri dishes (preformed gel treatment). For the two component treatment, alginate (0.5 mL) and Ca^2+^/chitosan mixtures (0.5 mL) were added at the same time on top of the glass slides. The preformed gels were first washed with PBS before being placed on the glass slides. The gels on the glass slides were incubated for three hours at 37 °C.

**Bacterial viability analysis:** Gels were taken off from the glass plates, and 30 µL of live/dead staining solution (BacLightTM molecular Probes Europe BV, Leiden, The Netherlands) was pipetted on top of the glass slides, and incubated in the dark for 30 min, after which the glass slides were immersed in PBS. Fluorescent microscopy (Leica DM 4000 B, Leica microsystems Heidelberg Gmbh, Heidelberg, Germany) was used to assess living and dead bacteria, and images were taken at least three random spots from each sample. Live/dead ratios were determined by using ImageJ (NIH, Bethesda, MD, USA). All experiments were performed in triplicate on three different days.

**Statistical analysis:** Data was tested for normality by using D’Agostino–Pearson and Shapiro–Wilk tests (*p* < 0.05). When data was distributed normally, an ANOVA with Tukey’s post-hoc test was performed, while in cases of non-normal distribution, a Kruskall–Wallis test was performed, followed by a Dunns test. All statistical analysis was performed using Graphpad Prism (Version 5.00, GraphPad Software, San Diego, CA, USA).

## 3. Results

Alginate is a negatively charged polymer at neutral pH, while chitosan is a positively charged polymer under mildly acidic conditions (p*K*_a_~6.1–6.6). Approximately 70% of the amino-groups within the chitosan polymer are charged at pH 6.0, which is reduced to 30% going to pH 7.0 [29]. Though, the overall protonation of chitosan decreases, still many positive charges are present and are able to interact with the negatively charged alginate, be it in a modest form. Exchanging gradually the conventionally used Ca^2+^ ions for chitosan would provide hydrogels with altered morphology and mechanical properties with additionally the anti-microbial properties of chitosan.

**Figure 1:**
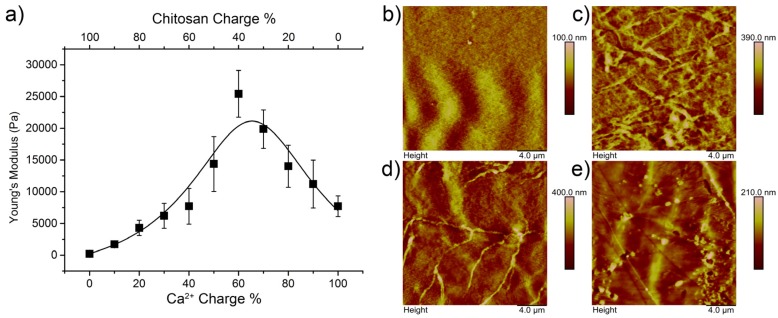
On the left a graph displaying the Young’s modulus dependent on the chitosan charge ratio (**a**); the line is added to guide the eye. Each point is an average of three measurements. On the right, representative AFM pictures of gels with a chitosan charge ratio of 0.0 (**b**), 0.2 (**c**), 0.4 (**d**), and 0.6 (**e**) are shown.

Alginate–chitosan hydrogels were prepared by mixing alginate solution (2 wt % in 0.15 M NaCl aqueous solution, pH 7.0) with the gelation solution in a 1:1 ratio. The gelation solution consisted of CaCl_2_ (50 mM aqueous solution, pH 8.0) and chitosan (1.5 wt % in 0.15 M NaCl aqueous solution, pH 6.0) in different volume ratios (Figure S1). It was found that a combination of Ca^2+^ and chitosan affected the mechanical properties of the hydrogel (Figure 1a). Changing the ratio of Ca^2+^:chitosan in a regular fashion going from Ca^2+^:chitosan-100:0 to Ca^2+^:chitosan-0:100, affected the stiffness in a non-linear fashion. A maximum stiffness of 26 kPa was achieved at a Ca^2+^:chitosan-60:40 charge ratio. Chitosan is not strongly charged at neutral pH and a minimum amount of 40% of charge from Ca^2+^ is needed to maintain appropriate cross-linking. Chitosan is able to coordinate to metal-ions through polar interactions although less strongly than alginate at neutral pH [30]. Nevertheless, the possibility of coordination of both alginate and chitosan most likely causes the increase in stiffness at a Ca^2+^:chitosan-60:40 charge ratio. Reducing the Ca^2+^ concentration below a charge fraction of 0.6 lowered the stiffness again. For the ratios, the assumption was made that both solutions of Ca^2+^ and chitosan used for gelation contain the same amount of positive charge (100 mM). However, it is clear that this is not maintained at pH 7 due to the p*K*_a_ of chitosan but it provides a clear approach for the established mixing procedure.

It was expected that the addition of chitosan and altering the Ca^2+^ concentration would alter the gel morphology. Alg–Ca^2+^:Ch-100:0, Alg–Ca^2+^:Ch-80:20, Alg–Ca^2+^:Ch-60:40, and Alg–Ca^2+^:Ch-40:60 were analyzed in the hydrated state using atomic force microscopy (AFM) to identify morphological changes. It was found that, compared to the chitosan-free gel (Alg–Ca^2+^:Ch-100:0) and the Alg–Ca^2+^:Ch-80:20 and Alg–Ca^2+^:Ch-60:40 gels showed increased fiber formation. Especially for the latter, long distinct fibers were observed, whereas gel fibers for the Ca^2+^:Ch-80:20 were shorter but higher in numbers. The Alg–Ca^2+^:Ch-40:60 gel did not show as many fibers as the other chitosan containing gels. The trend found for the stiffness of the gels could be correlated to the AFM analysis as chitosan induces fiber formation up until a point, after which that effect diminishes (Figure 1b–e, Figure S2).

For a potential wound dressing system involving hBM-MSCs as an active component to delegate the wound regeneration, the matrix should not influence cell viability. Viability was analyzed using XTT and complemented with a proliferation study combined with live/dead staining to determine not only the effect on cell density but also the fraction of alive and dead cells.

**Figure 2:**
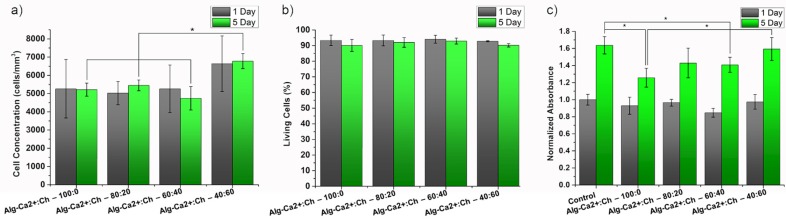
Results of the cell encapsulation experiments and cytotoxicity assessment of the gels. Cell concentration given in cells/mm^3^ of gels composed of alginate and different charge ratios of chitosan to Ca^2+^ for 1 and 5 days of culture (**a**). The percentage of living cells counted from confocal microscopy images for the same gels compared for 1 and 5 days (**b**). Representative images of encapsulated cells can be found in the Supplementary Materials (Figure S3). Results of the XTT assay of hBM-MSCs cultured together with gels of different compositions for 1 and 5 days (**c**). An asterisk indicates statistical significance (*p* ≤ 0.05).

For the viability assay (XTT), gels were prepared and incubated together with the hBM-MSCs for 1 and 5 days. It was found that none of the gels displayed any cytotoxicity after 1 day of exposure. For the 5 day exposure, slight decreases for the Alg–Ca^2+^:Ch-100:0 and the Alg–Ca^2+^:Ch-40:60 gels were observed with respect to the control, which was the used amount of alginate used for gel formation without the gelation solution (Figure 2c). The change in metabolic activity as indicated by the XTT assay could be a consequence of different hydrogel structures as the hydrogel density changes, allowing for better transport of soluble factors through the gels.

Furthermore, cells were observed while being encapsulated in the gels. Cells were stained using a live/dead viability kit to see if there is a cytotoxic effect upon encapsulation. Analysis showed that after 1 day of culture, cell densities were very similar to the seeding density (~10^6^ cells/mL in alginate). Only for the Alg–Ca^2+^:Ch-40:60 gel a slightly higher cell density was observed, which can be explained by contraction during gel formation leading to a lower gel volume (Figure 2a). Live/dead analysis showed no significant difference between the gels and was with a live percentage of >90%, regarded as non-toxic (Figure 2b). After 5 days, the cell concentration as well as the living ratio remained constant. This indicates that the gels do not kill the hBM-MSCs, but also do not support proliferation inside the gels.

In addition to alginate and chitosan not affecting hBM-MSC viability, chitosan is known to possess anti-microbial properties. As the future intended application would entail wound dressings and wound filling materials for regenerative medicine, the anti-microbial effect of the gels were tested towards two bacterial strains which have been isolated from wounds, namely *P. aeruginosa* and *S. aureus*. Both pose as a high medical risk as *P. aeruginosa* is an opportunistic nosocomial pathogen and *S. aureus*, of which methicillin-resistant *S. aureus* (MRSA) is the most well-known, is becoming a major global threat [27,28].

It was found that the addition of chitosan had a significant anti-bactericidal effect on both *P. aeruginosa* and *S. aureus*. Both the preformed hydrogels and wound-filling model of Alg–Ca^2+^:Ch-100:0, Alg–Ca^2+^:Ch-80:20, Alg–Ca^2+^:Ch-60:40, and Alg–Ca^2+^:Ch-40:60 were tested and compared to a PBS control. Glass slides were covered with bacterial suspension and rinsed after a 1 h incubation time for sufficient surface attachment. Either a preformed hydrogel rinsed with PBS was placed on top of the bacteria-coated glass slides, representing a wound dressing model, or the hydrogels were directly formed on top of the glass substrates simulating a wound filling model. Incubation with the hydrogels was done for 3 h after which the live/dead analysis was performed determining the reduction of living bacteria on the glass slide.

**Figure 3:**
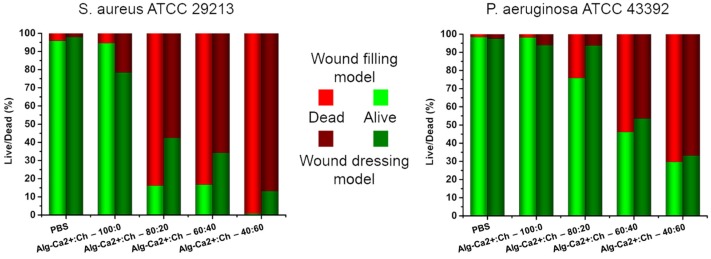
Bactericidal experiments of chitosan containing hydrogels. Gels with a different positive charge ratio of chitosan and Ca^2+^ were tested using two different methods. Representative pictures of *S. aureus* and *P. aeruginosa* adhered on glass and treated with chitosan containing hydrogels are shown together with a corresponding ratio of dead and alive bacterial cells. Red indicates the amount of dead bacteria while green represents alive bacteria. Light colors show results for direct mixing (wound filling model) and dark colors for the pre-formed gels (wound dressing model). Representative pictures of bacteria can be found in the Supplementary Materials (Figures S3 and S4).

In both models, the bactericidal effect towards *S. aureus* was higher than towards *P. aeruginaosa*. It was found that gels with higher chitosan fractions were more effective than the ones with lower chitosan fractions. The dressing model of Alg–Ca^2+^:Ch-80:20, Alg–Ca^2+^:Ch-60:40, and Alg–Ca^2+^:Ch-40:60 gave a reduction of 58%, 66%, and 87% of living bacteria for *S. aureus* and 6%, 46%, and 67% for *P. aeruginosa*. Compared to PBS and Alg–Ca^2+^:Ch-100:0, *P. aeruginosa* was similarly affected by the Alg–Ca^2+^:Ch-80:20 all having a negligible reduction in living bacteria (Figure 3). Using the wound filling model, a higher bactericidal effect was observed as for the dressing model. The filling model of Alg–Ca^2+^:Ch-80:20, Alg–Ca^2+^:Ch-60:40, and Alg–Ca^2+^:Ch-40:60 gave a reduction of 84%, 83%, and >99% of living bacteria for *S. aureus* and 24%, 54%, and 70% for *P. aeruginosa* while PBS control reduced the amount of living bacteria only marginally (Figure 3). The enhanced effect of the Alg–Ca^2+^:Ch-40:60 compared to the Alg–Ca^2+^:Ch-60:40 is probably caused by both the higher concentration and the enhanced mobility of the chitosan due to the lower modulus. This higher activity due to enhanced mobility is supported by comparing the same chitosan fraction between the different models (filling versus dressing). Initially, chitosan mobility is high for the wound filling model and low for the dressing model.

## 4. Conclusions and Discussion

The developed alginate–chitosan system displays a dual functionality important for the purpose of wound healing. It enables encapsulation of hBM-MSCs in an efficient way without reducing their viability, and the chitosan functions as an anti-microbial agent tested against two major strains involved in wound infections. The two-component system offers the possibility of using it either as a preformed dressing or as a wound filling model. The wound filling model offers two advantages: (1) using two liquid components allows sufficient contact with the whole wound before setting as a gel; (2) it has a higher bactericidal effect. In particular, wounds that remain non-healing, e.g., diabetic ulcers in need of debridement, would potentially benefit from this system as it is known that stem cells enhance wound healing, and infection is often a major complication [4,9,31,32,33]. The preformed hydrogels have the advantage of being stored for long periods of time. Alginate is often used as a storage medium for frozen (stem) cells and a preformed wound dressing stored in a frozen state would allow mass production and having the dressing ready at hand. There are already commercially available products on the market based on either alginate or chitosan such as Cheerain^®^ alginate patches and HemCon^®^ chitosan-coated bandages for the purpose of wound dressing but not combined and not for the purpose of additionally containing mesenchymal stem cells. There are systems known where chitosan and alginate have been combined but often as separated layers, as polyelectrolyte coatings on non-hydrogel substrates or as hydrogel formed at relatively low pH. The latter approach requires various drying and washing steps and is therefore not compatible with encapsulated cells [7,34,35,36]. Hence, translation towards clinical use of the proposed system is regarded as very promising, as the materials are already used for these specific applications, can include stem cells, and can be applied as both a preformed dressing and a two-component mixing system.

## Supplementary Materials

The following are available online at www.mdpi.com/2073-4360/9/4/149/s1. Figure S1: Atomic Force Microscopy analysis of hydrogels; Figure S2: hBM-MSC live/dead imaging encapsulated in gels; Figure S3: *P. aeruginosa* live/dead imaging; Figure S4: *S. aureus* live/dead imaging.
