# Supplementary material for: Anti-Microbial Biopolymer Hydrogel Scaffolds for Stem Cell Encapsulation
Source: Polymers (Basel). 2017 Apr 22;9(4):149. doi: 10.3390/polym9040149 (PMC6431895; doi:10.3390/polym9040149)
Supplement: Supplementary file 1 [file polymers-09-00149-s001.pdf]

Supplementary Materials: Anti-Microbial Biopolymer Hydrogel Scaffolds for Stem Cell Encapsulation

Philipp T. Kühn, René T. Rozenbaum, Estelle Perrels, Prashant K. Sharma and Patrick van Rijn

CONTENTS:

S1 Atomic Force Microscopy analysis of hydrogels
S2 hBM-MSC live/dead imaging encapsulated in gels
S3 *P. aeruginosa* live/dead imaging
S4 *S. aureus* live/dead imaging

S1 Atomic Force Microscopy analysis of hydrogels

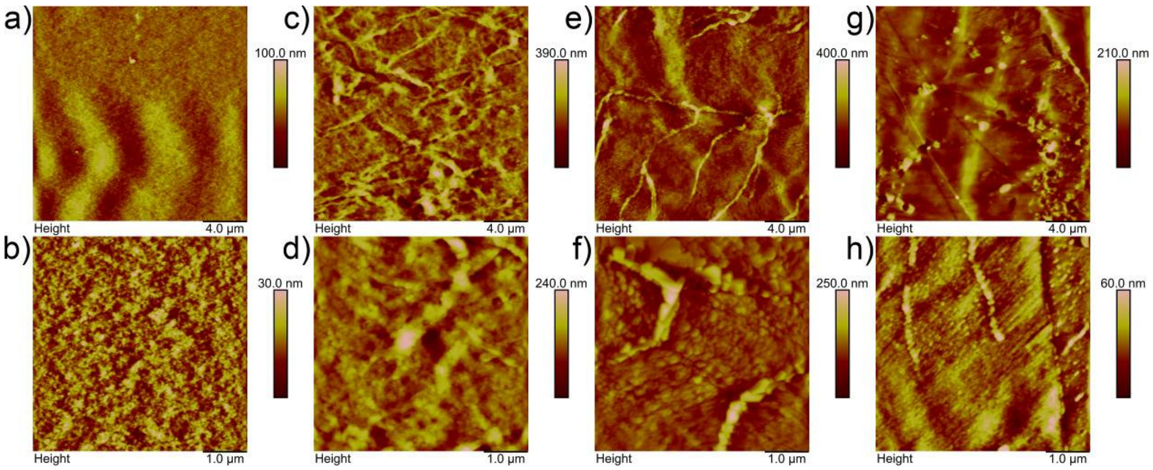

Figure S1: Atomic force microscopy images of the Alg–Ca^{2+}/Ch—(100:0) (**a** and **b**), Alg–Ca^{2+}/Ch—(80:20) (**c** and **d**), Alg–Ca^{2+}/Ch—(60:40) (**e** and **f**) and Alg–Ca^{2+}/Ch—(40:60) (**g** and **h**). For each gel composition, two pictures are shown in different sizes.

S2 hBM-MSC live/dead imaging encapsulated in gels

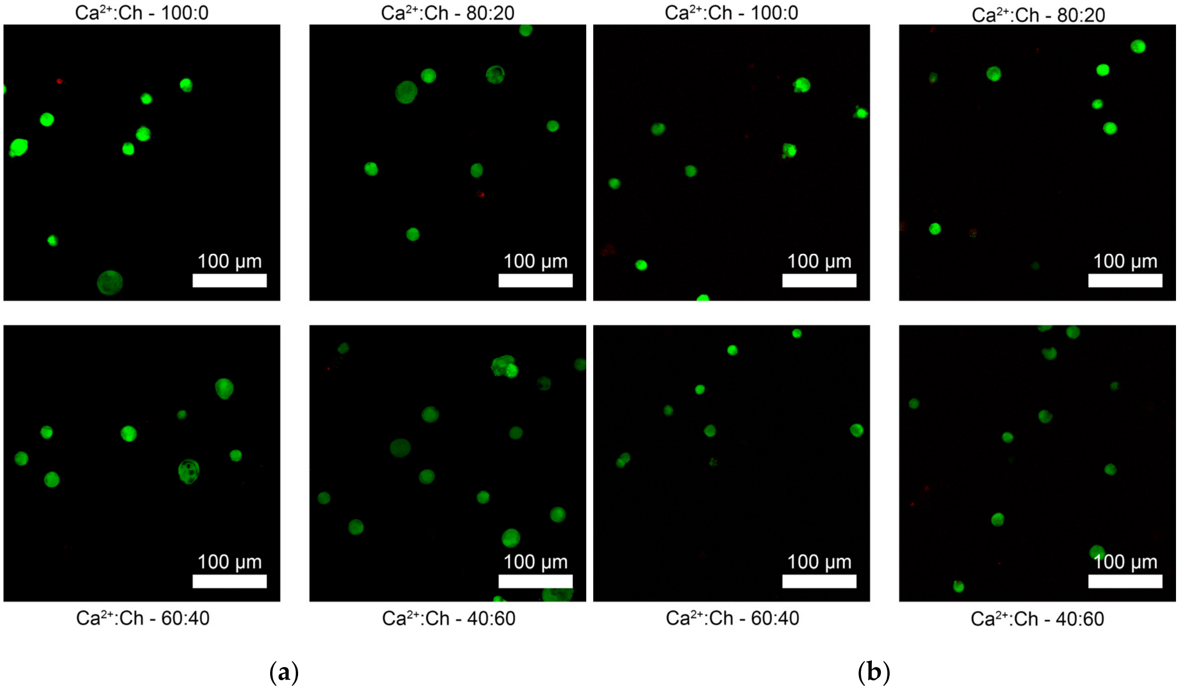

Figure S2: Representative pictures of the live/dead staining of encapsulated hBM-MSCs in hydrogels of all compositions after 1 day (**a**) and 5 days (**b**) of culturing. Green cells are living, the nucleus of dead cells is stained red. Measuring depth was 50 µm for all pictures.

S3 *P. aeruginosa* live/dead imaging

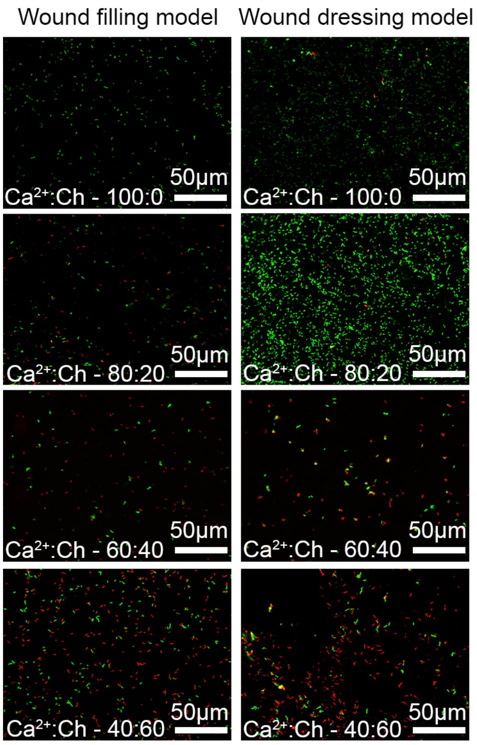

Figure S3: Representative pictures of the live/dead staining of *P. aeruginosa* cells adhered to glass and treated with gels of displayed composition using the wound filling (left) and wound dressing (right) approach. Living cells are stained in green and dead cells are stained in red.

S4 *S. Aureus* live/dead imaging

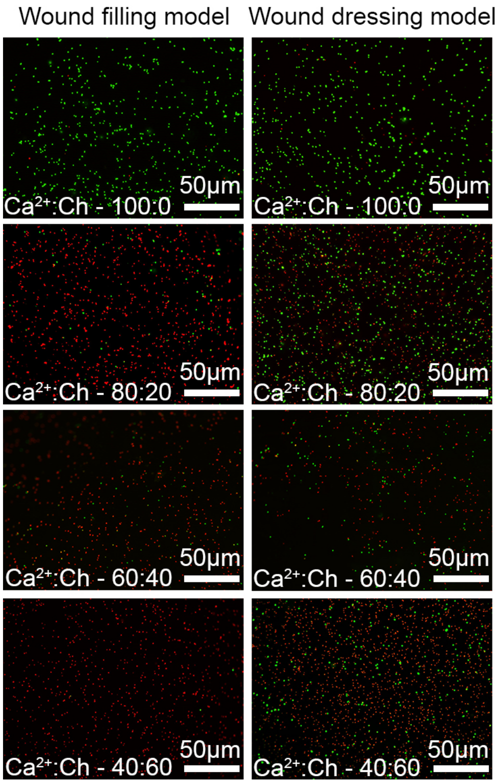

Figure S4: Representative pictures of the live/dead staining of *S. aureus* cells adhered to glass and treated with gels of displayed composition using the wound filling (left) and wound dressing (right) approach. Living cells are stained in green and dead cells are stained in red.
